# Two decades of growth and trends in the FDA authorization of digital medical devices

**DOI:** 10.1038/s41746-026-02692-5

**Published:** 2026-06-17

**Authors:** Alexander O. Everhart, Cirrus Foroughi, Melissa Ouellet, Ariel D. Stern

**Affiliations:** 1 Division of General Medicine and Geriatrics, John T. Milliken Department of Medicine, Washington University School of Medicine in St. Louis, St. Louis, MO USA; 2 Center for Advancing Health Services, Policy & Economics Research, Washington University in St. Louis, St. Louis, MO USA; 3 Berkeley Research Group, Boston, MA USA; 4 Hasso Plattner Institute, University of Potsdam, Potsdam, Germany; 5 Department of AI and Human Health, Icahn School of Medicine at Mount Sinai, New York, NY USA

**Keywords:** Business and industry, Engineering, Health care, Medical research

## Abstract

The regulatory environment for digital medical devices has rapidly changed in recent years as policymakers have worked to keep up with the evolving landscape of biomedical technologies. Despite the need for new regulatory efforts, existing data assets in the US are not capable of systematically tracking whether FDA-authorized medical devices have digital components, limiting regulators’ ability to incorporate software-specific considerations into post-market surveillance activities and limiting other stakeholders’ ability to understand the extent of digitization of different medical specialty areas and product categories. We pioneer a new application of text analysis, using records from tens of thousands of regulatory documents for newly authorized medical devices, to describe the digital transformation of the US medical device industry over the past two decades. We show that the number of medical devices with digital components has grown substantially over time, with meaningful heterogeneity across clinical specialties.

## Introduction

Recent years have seen significant progress in the regulation of digital medical devices. In the United States, this has involved the establishment of the FDA’s Digital Health Center of Excellence^1^, the launch of new regulatory initiatives related to cybersecurity^2^ and virtual reality^3^, the publication of over twenty regulatory guidance documents with digital health content^4^, and a regularly-updated list of Artificial Intelligence/Machine Learning (AI/ML)-Enabled Medical Devices^5^.

These developments represent timely progress towards modernizing medical device regulation for an increasingly digital and software-reliant set of products, yet historical regulatory databases remain ill-suited for understanding the digital transformation of the device industry. Existing FDA regulatory databases do not denote whether a device has software components, let alone distinguish between whether a device is a standalone software product (Software as a Medical Devices, SaMD) vs. when it represents a combination product with both digital and analog components (Software in a Medical Device, SiMD) or other relevant nuances^6^.

We present newly collected data to document the digital transformation of the U.S. medical device industry over recent decades. Using data scraped from FDA product summaries over 20 calendar years, we describe and characterize the increasing digitization of newly authorized devices and differences across medical specialty areas.

## Results

### Study sample

Our study sample included 47,712 medical devices authorized over the calendar years 2005 through 2024 (inclusive), spanning 1,514 product codes and brought to market by 9,952 unique firms (Table 1). 194 (0.4%) devices were cleared through the de Novo pathway; the remaining 47,518 (99.6%) devices were cleared through the 510(k) pathway. Overall, 13,390 devices (28.1% of the sample) included “software” as a keyword in regulatory documents (hereafter referred to as “digital medical devices”), of which 921 (1.9%) were classified as SaMD and 12,469 (26.1%) as SiMD. The extent to which medical devices included software varied widely across clinical specialties, ranging from 6.5% of orthopedic devices (*n* = 671) to 76.5% of radiology devices (*n* = 5627).

**Table 1:** Characteristics of Authorized Medical Devices

	All^a^	Cardiovascular	Clinical Chemistry	Dental	Gastroenterology and Urology	General Hospital	General, Plastic Surgery	Orthopedic	Radiology
*N*	47,712	6811	2512	4814	3068	5921	6844	10,388	7354
Unique product codes	1514	224	245	142	257	195	265	174	124
Unique firms	9952	1528	443	1365	862	2225	2082	1190	1617
De Novo, *N* (%)	194 (0.4%)	33 (0.5%)	25 (1.0%)	6 (0.1%)	40 (1.3%)	18 (0.3%)	40(0.6%)	16(0.2%)	16(0.2%)
Implantable device, *N* (%)	14,001 (29.3%)	353 (5.2%)	2 (0.0%)	1,819 (37.8%)	499 (19.5%)	221 (3.7%)	1,318(19.3%)	9,721(93.6%)	68(0.9%)
Digital device, *N* (%)	13,390 (28.1%)	2,284 (33.5%)	956 (38.1%)	678 (14.1%)	655 (21.3%)	683 (11.5%)	1,836(26.8%)	671(6.5%)	5627(76.50%)
Software as a medical device^b^, *N* (%)	921 (1.9%)	159 (2.3%)	26 (1.0%)	17 (0.4%)	10 (0.3%)	14 (0.2%)	5(0.1%)	9(0.1%)	681(9.3%)
Software in a medical device^b^, *N* (%)	12,469 (26.1%)	2,125 (31.2%)	930 (37.0%)	661 (13.7%)	645 (21.0%)	669 (11.3%)	1,831 (26.8%)	662(6.4%)	4946(67.3%)
New digital product code^c^, *N* (%)	246 (0.5%)	46 (0.7%)	34 (1.4%)	16 (0.3%)	36 (1.2%)	28 (0.5%)	38 (0.6%)	18(0.2%)	30(0.4%)
First digital product in existing product code^d^, *N* (%)	356 (0.7%)	48 (0.7%)	62 (2.5%)	37 (0.8%)	61 (2.0%)	36 (0.6%)	55 (0.8%)	42(0.4%)	15(0.2%)

^a^Sample includes all medical devices in the top 8 medical specialties authorized between 2005 and 2024.

^b^“Software as a medical device” and “Software in a medical device” were identified based on a keyword search within the regulatory authorization document. SaMD was identified using the following keywords: “Software as Medical Device,” “Software as a Medical Device,” “Standalone software,” “Software only,” and “SaMD.”

^c^“New digital product code” denotes when a device is both the first medical device in a product code and has a digital component (i.e., natively digital” devices).

^d^“First digital product in existing product code” denotes when a device is not the first medical device in a product code but is the first to include a digital component within the product code (i.e., “digitization”).

While over approximately one quarter of the sample was comprised of digital medical devices, only a small minority of these devices represented new technologies: 246 (0.5%) devices were both digital devices and the first cleared device within their product code, and 356 (0.7%) were the first digital device in a previously analog product code. This implies that the vast majority of software devices were cleared after other software devices with similar indications and mechanisms of action (i.e., in the same product code) had already received FDA clearance.

### Trends in digital medical device clearances

**Fig. 1:**
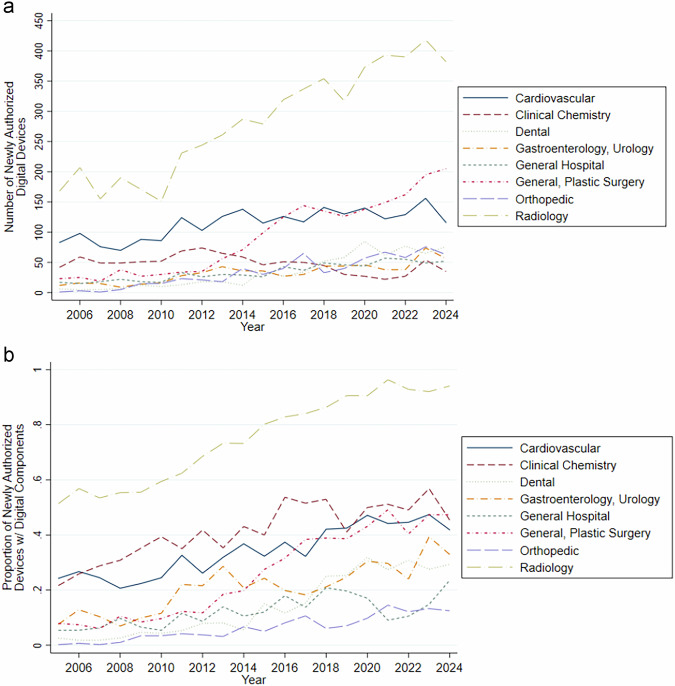
Trends in digital device volume by clinical specialty. Sample includes all medical devices in the top 8 medical specialties authorized between 2005 and 2024.

The number of digital medical devices cleared annually has grown substantially over time. With the exception of clinical chemistry, all clinical specialties studied had more software devices authorized in 2024 vs. 2005 (Fig. 1a). However, there were differences in growth rates by clinical specialties. For example, there were 382 radiology devices with software cleared in 2024 vs 168 in 2005, representing a 227% increase. In contrast, there were just 35 clinical chemistry devices with software cleared in 2024 vs. 42 in 2005. Digital medical devices have also grown to represent a larger share of all cleared medical devices. The proportion of devices with software grew for all clinical specialties studied (Fig. 1b). In 2024, four of the eight clinical specialties studied had software in over 40% of their cleared devices, as compared to just one in 2005.

**Fig. 2:**
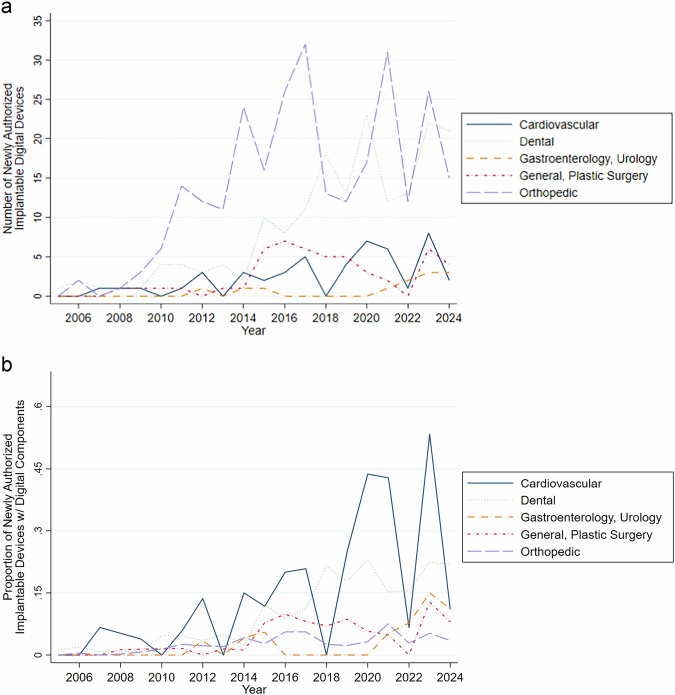
Trends in digital device volume by clinical specialty and implantable status. Notes. Sample includes implantable medical devices in Cardiovascular, Dental, Gastroenterology and Urology; General, Plastic Surgery; and Orthopedic specialties authorized between 2005 and 2024. Medical devices from Clinical Chemistry, General Hospital, and Radiology are omitted for visual clarity.

Software was increasingly incorporated into implantable medical devices during the sample period, albeit with less pronounced growth compared to the overall study sample (Fig. 2a). In 2024, proportions of devices with digital components and/or use attributes ranged from 3.5% of implantable orthopedic devices to 21.9% of implantable dental devices (Fig. 2b).

**Fig. 3:**
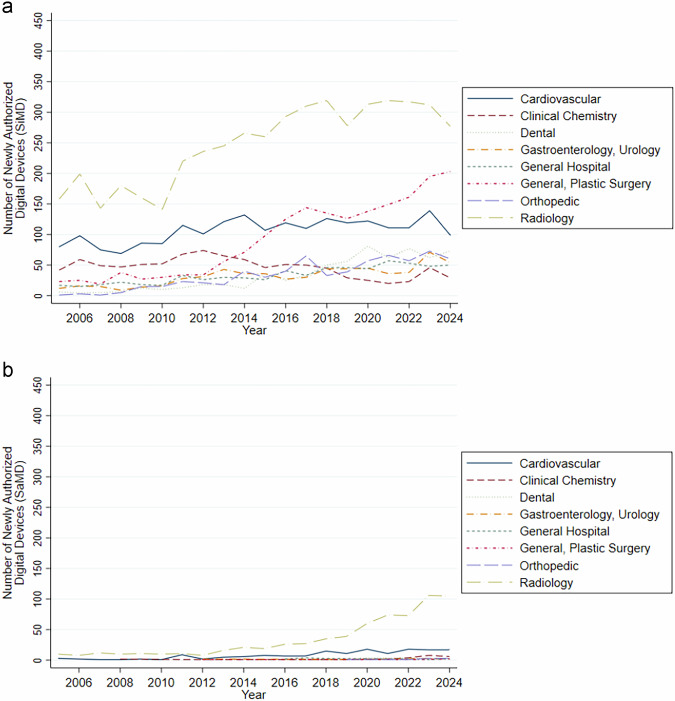
Trends in digital device volume by clinical specialty and software type. Notes. Sample includes all medical devices in the top 8 medical specialties authorized between 2005 and 2024. “Software as a medical device” and “Software in a medical device” identified based on a keyword search within the regulatory authorization document. SaMD was identified using the following keywords: “Software as Medical Device,” “Software as a Medical Device,” “Standalone software,” “Software only,” and “SaMD”.

Overall growth in digital medical devices was primarily driven by SiMD—i.e., combination software-hardware products. Similar to the patterns in digital devices generally, all studied clinical specialties (except clinical chemistry) had more SiMDs authorized in 2024 than in 2005 (Fig. 3a). Conversely, only two clinical specialties, cardiovascular and radiology, ever had more than 10 SaMD—i.e., stand-alone software devices—cleared within a given calendar year (Fig. 3b). In both SiMD and SaMD, radiology regularly had the most medical devices cleared in a given year.

**Fig. 4:**
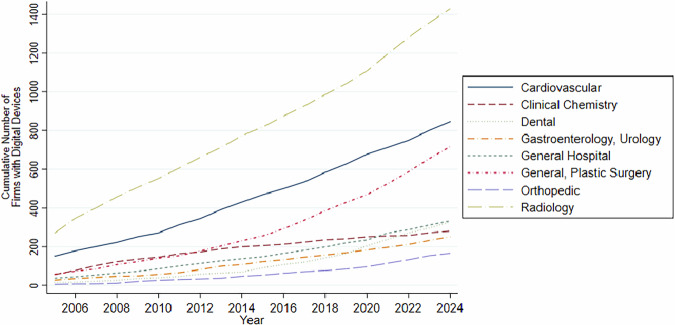
Cumulative number of firms with authorized digital devices by clinical specialty. Sample includes all firms with authorized medical devices in the top 8 medical specialties between 2005 and 2024.

In general, new manufacturers of digital medical devices continued to enter the market and receive FDA clearances (Fig. 4), but the number of firms varied across specialties. For example, by 2024, a total of 1,429 unique manufacturers received clearance for at least one digital radiology device, and 166 unique manufacturers had received clearance for at least one digital orthopedic device. However, growth in the cumulative number of firms appears largely linear over time, with the exception of general and plastic surgery, where the number of firms developing digital devices within the specialty has grown more rapidly.

**Fig. 5:**
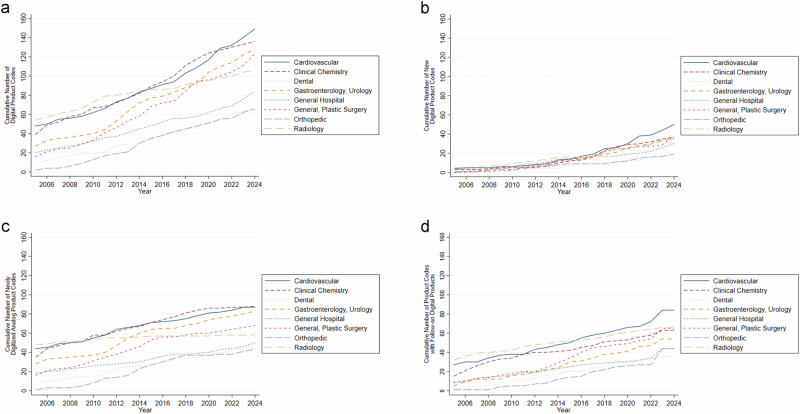
Trends in digital device product types by clinical specialty. Sample includes all medical devices in the top 8 medical specialties authorized between 2005 and 2024. “New digital product code” denotes when a device is both the first medical device in a product code and has a digital component (i.e., natively digital” devices). “Digitized analog product codes” denotes when a device is not the first medical device in a product code but is the first to include a digital component within the product code.

The number of different types of digital medical devices has also increased over time. The cumulative number of product codes—device categories that capture a specific context of use and function^7^—with at least one software device has increased on a largely linear basis for many clinical specialties (Fig. 5a), indicating the creation and authorization of new types of digital devices. Increases in the number of product codes with software were driven by a combination of the creation of new and digital product codes (i.e., the creation and authorization of devices that always appear with software, or are natively digital) (Fig. 5b) and the digitization of existing analog product codes (i.e., the authorization of devices that add a new digital component to a previously analog device (Fig. 5c), with the cumulative number of digitized product codes generally exceeding the number of natively digital product codes in most clinical specialties and in most years. The relative rankings of which clinical specialties had the most cumulative digital product codes were largely similar when looking at either natively digital product codes or subsequently digitized product codes.

More manufacturers have also developed a wider range of medical devices over time. The number of product codes with cleared digital devices from two or more manufacturers has increased over time across all studied clinical specialties (Fig. 5d). However, counts of product codes with digital devices from two or more manufacturers in a given year and clinical specialty were often substantially lower than the total number of digital product codes in the same year and specialty (Fig. 5a), implying that many digital devices are only made by a single manufacturer.

## Discussion

In this study of medical devices authorized in the United States over the years 2005–2024, we observed evidence of the digitization of the medical device industry. The annual number of devices with software has grown over time, covering complex medical technologies, including implantable devices, with these devices coming from a growing number of manufacturers. The number of different digital product types, including both SaMD and SiMD, has increased, representing a growing number of types of medical technologies incorporating software. However, the pace of growth in SiMD has largely outpaced SaMD, consistent with prior work showing that growth in SaMD specifically has been relatively slow^8^.

Our study highlights that while the medical device industry has generally incorporated more software into its products, digitization is not a monolithic process. Some specialties have focused more on developing native digital devices. For example, radiology continued to predominantly develop new digital products over the period studied, with less emphasis on digitizing previously analog devices. These new and natively digital devices included clinically innovative technologies that could not feasibly exist without a software component, such as digital mammography systems^9^ and computer-assisted detection software used to identify concerns in medical images^10^. In contrast to radiology, other specialties consistently developed both new digital products and continued to develop digital versions of existing analog product types. For example, during the study period, newly authorized cardiology devices included implantable cardiac arrhythmia detectors^11^ (a natively digital approach) or electrocardiograms augmented with new algorithms (representing the addition of new software to an existing product)^12^. These differences in digitization processes across specialties may reflect underlying clinical opportunities. It may be that most radiology devices without software are either no longer relevant or will never need software, while cardiology still presents opportunities to develop new technologies and add digital features to existing products. These differences may also reflect differences in regulatory requirements, reimbursement environments, or underlying costs or market structures – all of which warrant further study.

Importantly, the growth in digital medical devices over time should not be interpreted as evidence that the market for such devices has become saturated. While the number of firms with cleared digital devices has grown over time, the count of products with two or more manufacturers with an authorized digital medical device as of 2024 was roughly 40% of the total number of digital products. Put differently, over half of the digital medical devices authorized as of 2024 had only one manufacturer developing the product. Opportunities still exist for competitors and differentiated products to enter the market for many types of digital devices.

Our findings generally speak to the growing need for a comprehensive data infrastructure to support digital device regulation and development. In contrast to AI-enabled medical devices, which are systematically documented by the FDA^5^, the FDA does not maintain public databases that document software inclusion/components for non-AI devices. Regulators will need to develop new and better tools for their colleagues as well as patients, procurement organizations, and providers to track these devices in a systematic way, rather than analyzing individual product descriptions and summary documents (as done in our study). This need is particularly urgent, as new coding tools such as large language models may increasingly facilitate the development of new clinical software and further accelerate the development of new product modules, features, and the digitization of the device industry more broadly^13,14^. Furthermore, prior work has found that digital medical devices experience unique safety issues and more adverse events and recalls compared to both devices without software and AI-enabled medical devices specifically^15–18^. Ongoing staffing reductions and reorganizations within the FDA^19^ underscore the need to collect and publish timely and relevant data in order to reduce burdens associated with post-market surveillance and other activities.

In addition to policy changes, our work also highlights the importance of further developing digital health literacy and training in medical and nursing degrees and continuing medical education^20^. Clinicians regularly report that they are not sufficiently prepared to safely and effectively deploy digital technologies^21–24^, while most medical schools do not include digital health training in their curricula^25–27^. Yet the tools clinicians use are becoming increasingly digital. Efforts to standardize routine digital medical literacy and training for clinicians may improve the effective use of digital medical devices.

There are several limitations to our study. First, we identified digital medical devices with a natural language processing tool applied to regulatory documents provided by the FDA. To the extent that software capabilities were not fully described or disclosed within these documents, our results may reflect undercounts of digital medical devices. Second, we identified new digital products as well as the digitization of previously analog products based on a history of prior software devices. Our main study period spanned 2005 to 2024, with data from 2002 to 2004 used exclusively to make historical comparisons. To the extent that certain types of medical devices have a greater than three-year lag between product authorizations by any manufacturer, we may have incorrectly categorized digitized versions of previously analog products as new digital devices. Third, our study only focused on the top eight clinical specialties for 510(k) and de Novo devices. While 510(k) and de Novo devices make up over 99% of devices authorized by the FDA, and our studied clinical specialties represented the large majority of 510(k) and de Novo devices authorized during the study period, our findings may not be generalizable to 510(k) and de Novo devices from other (smaller) clinical specialties, devices authorized through the PMA pathway for high-risk devices, devices exempt from traditional evidence requirements as part of the Humanitarian Device Exemption pathway, low-risk devices or devices subject to enforcement discretion that do not require premarket review, or clinical decision support software that is not regulated as a medical device.

In conclusion, the number of medical devices with software authorized within the US has grown substantially over time, both through the development of new digital technologies and the digitization of previously analog technologies, with meaningful heterogeneity across different clinical specialties. However, the market for digital medical devices is far from saturated or competitive: there are many cases where only one manufacturer has received FDA authorization for a given type of digital device. More work is needed to develop the data infrastructure required to track and evaluate these devices as they represent a growing share of the medical devices available to patients and providers.

## Methods

### Data sources

This cross-sectional analysis was performed using two main data sources. First, we relied on public medical device authorization databases from the FDA, which provide a census of all medical devices authorized by the FDA since 1976^28,29^. These databases included standardized identifiers that describe the regulatory pathway used to authorize devices, along with device names, manufacturers, clinical specialties, and “product codes” that describe the generic function of the device as well as whether a device is implantable^7^. Starting in 2002, the FDA’s authorization databases also regularly included non-standardized “summary documents”, wherein manufacturers described device functions, indications, and performance characteristics. These databases have been used extensively in prior studies^15–17,30–35^.

Second, we identified software in FDA-authorized medical devices using Nyquist MedTech, a database derived by applying optical character recognition techniques and natural language processing algorithms to FDA authorization records^36^. Nyquist MedTech augments the FDA authorization databases by making non-standardized summary documents machine-searchable, meaning specific words and phrases within authorization records can be systematically identified. Nyquist MedTech provides a novel, streamlined platform for identifying words and phrases, but prior work has used similar techniques to extract information from FDA records^8,15,37–40^.

This study used publicly available materials and did not involve humans. Therefore, ethics committee approval was not required.

### Study sample

The study sample included medical devices from the top eight clinical specialties (ranked based on total FDA clearances) cleared through the 510(k) and de Novo pathways between January 1, 2005, and December 31, 2024, as identified in the FDA authorization databases^28^. The eight largest clinical specialties were: cardiovascular, clinical chemistry, dental, gastroenterology and urology, general hospital, general and plastic surgery, orthopedic, and radiology. Sample sizes associated with different inclusion criteria are presented in Table S1 in Supplementary Data 1. The focal eight clinical specialties accounted for 75.6% of all 510(k) and de Novo devices authorized during the study period. Among devices in the top eight clinical specialties, 98.8% had machine-readable summary files.

The de Novo and 510(k) pathways are the primary mechanisms used by the FDA to review Class II devices. Devices are designated as Class II when following best manufacturing practices is insufficient for ensuring the safety and effectiveness of the device, but human clinical evidence is not necessarily required. 99% of medical devices reviewed by the FDA are considered Class II, and these range in complexity from surgical gloves to knee replacements. Class I devices are considered low enough risk such that FDA review is not warranted (e.g., surgical trays), while Class III devices require human clinical evidence to support their approval (e.g., cardiac pacemakers)^41^.

Class II devices are cleared through the de Novo pathway when the FDA has not previously authorized a device with a similar indication and mechanism of action, but the device is of sufficiently low risk to not warrant a Class III designation. De Novo devices are often, but not always, cleared on the basis of human clinical data^42^. In contrast, the 510(k) pathway is used for Class II devices where the FDA has previously authorized a device with a similar indication and mechanism of action. In this case, manufacturers demonstrate that their new device is “substantially equivalent” to a “predicate” with a similar indication and mechanism of action; substantial equivalence is typically evaluated via bench testing and rarely through human clinical data^41^. As Class II devices, both de Novo and 510(k) devices are identified in a single FDA database^28^.

### Identifying software status in medical devices

Digital devices, whether they were physical (hardware) devices with software components (SiMD), such as implantable cardiac monitors with arrythmia detection algorithms, or were comprised entirely of software (SaMD), such as cognitive behavioral therapy apps, were identified using Nyquist MedTech^36^. We first identified if medical devices included any software by performing a non-case-sensitive search within Nyquist MedTech for whether their summary documents included the word “software.” Manufacturers are not permitted to include extraneous text unrelated to describing device characteristics in product summary documents, meaning keywords such as “software” will not typically appear in product summaries unless they relate to aspects of the device’s functionality. A manual review of ten randomly selected devices within each calendar year flagged as including software (200 devices with software flags) and ten randomly selected devices within each year not flagged as including software (200 devices without software flags) yielded a 94% accuracy rate in identifying digital medical devices. False positives occurred when manufacturers specifically noted that their device did not contain software, and false negatives occurred when manufacturers described their devices using less common terms without using “software” (e.g., “microprocessor”).

We then assessed whether digital medical devices were considered to be SaMD by performing a non-case-sensitive search within Nyquist MedTech for the following terms: “SaMD”, “Software as Medical Device”, “Software as a Medical Device”, “Standalone software”, and “Software only”. We considered devices with any of these keywords to be SaMD, while software devices without SaMD keywords were considered to be SiMD.

### Outcomes

We analyzed several outcomes of interest on an annual basis to describe the different ways software may be introduced into medical devices over time. First, we calculated the annual total count of software devices cleared by the FDA during our study period, as well as the annual proportion of all devices cleared during our study period that included software. We calculated counts and proportions of software devices both for our entire sample and among implantable devices specifically. Second, we calculated separate annual totals of cleared SiMD and SaMD. Third, we calculated the cumulative number of manufacturers that had at least one cleared digital medical device. Manufacturer names were standardized (e.g., removal of extraneous punctuation) as described in prior work^15^.

Fourth, we calculated the cumulative number of “product codes” that included at least one software device. All medical devices are assigned a single product code, a standardized identifier that describes the generic function of a device. Examples include “Stent, Coronary,” “Stent, Carotid”, and “Stent, Renal”. These product codes are specific with respect to indication and mechanism of action, but do not necessarily differentiate between digital and non-digital medical devices. As such, product codes may be “natively” digital and only include software devices, entirely analog and never include software devices, or transition from analog to digital as a newly cleared device is the first to include software within the product code. The cumulative number of product codes with at least one software device describes the span of different types of devices that include software.

Fifth, we calculated the cumulative number of new and digital product codes. These counts describe product codes where the first authorized device within the product code included software and were first introduced during our study period, meaning no comparable devices with similar indications or mechanisms of action had received authorization from the FDA. Put differently, these are entirely new products that inherently include software as part of their design, such as a digital mammography system (product code “MUE”)^9^.

Sixth, we calculated the cumulative number of newly “digitized” product codes. These counts describe the number of product codes that previously included only non-software devices, but then a software device was authorized within the product code. Digitized product codes represent the digitization of existing device types, rather than the development of truly new products, such as the addition of a digital readout to a blood pressure cuff (product code “DXQ”)^43^. Seventh and finally, we calculated the number of product codes that included software devices from two or more manufacturers.

For all cumulative count measures, we considered data on devices cleared from 2002 to 2004 in addition to data on devices cleared during the study period, 2005-2024, in order to establish a sufficient history within a product code. For example, in order to differentiate whether a software device was the first software device within a product code, we examined the time period between 2002 and the clearance date for the device to see whether prior devices within the product code included software. We used this lookback period because the FDA authorization databases do not reliably include product summary documents for devices authorized before 2002. Consequently, we could not reliably identify whether medical devices included software prior to 2002. This would mean, for example, that for a software device cleared by the FDA on January 1, 2002, we could not reliably assess whether the device was the first software device.

### Statistical analysis

Outcomes of interest were calculated annually. All outcomes were calculated in strata across the eight different clinical specialties included in the study sample; outcomes stratified by clinical specialty were then plotted over time.

## Supplementary information

Supplement 1 - npj DM - Jan 2026.

## Data Availability

The data that support the findings of this study are available from the corresponding author upon request. Additionally, interested researchers may contact Nyquist AI for access requests to the Nyquist MedTech database (https://www.nyquistai.com/pricing).

## Code availability

The code that supports the findings of this study is available from the corresponding author upon request, including analysis code and code for performing device identifier-level linkages between public FDA data and Nyquist MedTech data assets.
