# Supplementary material for: Two decades of growth and trends in the FDA authorization of digital medical devices
Source: NPJ Digit Med. 2026 Jun 17;9:474. doi: 10.1038/s41746-026-02692-5 (PMC13282478; doi:10.1038/s41746-026-02692-5)
Supplement: Supplementary file 1 — Supplement 1 - npj DM - Jan 2026. [file 41746_2026_2692_MOESM1_ESM.docx]

**LIST OF SUPPLEMENT DATA 1 FIGURES AND TABLES IN ORDER OF APPEARANCE**

**Table S1:** Sample Construction Steps

**Table S1:** Sample Construction Steps

| **Step Description** | Number of Devices |
| --- | --- |
| All devices, 2002 to 2024 | 72,132 |
| Top 8 Specialty Devices, 2002 to 2024 | 55,942 |
| Top 8 Specialty Devices with machine-readable summary files, 2002 to 2024 | 55,284 |
| Top 8 Specialty Devices with machine-readable summary files, 2005 to 2024 | 47,712 |
